# 205. Utilization of the Four Themes of the CDC/ATSDR SVI Interactive Map in Assessing Initial Clostridioides difficile Infection Outcomes

**DOI:** 10.1093/ofid/ofae631.063

**Published:** 2025-01-29

**Authors:** Timothy Afable, Anna Zhou, Karen Tan, Jacinda C Abdul-Mutakabbir

**Affiliations:** Loma Linda University Health, Loma Linda, CA; Loma Linda University Medical Center, Loma Linda, California; Loma Linda University Medical Center, Loma Linda, California; University of California San Diego, San Diego, CA

## Abstract

**Background:**

Social determinants of health (SDoH) (e.g., education, clean water, health care access) are external factors shown to impact health outcomes. Inequities in SDoH have been hypothesized to contribute to poorer *Clostridioides difficile* infection (CDI) outcomes. The Centers for Disease Control’s (CDC) Social Vulnerability Index (SVI) is a composite measure of neighborhood (census tract) based on four major themes: socioeconomic status (SES), housing characteristics (H&C), race/ethnicity (REM) status, and housing and transportation (H&T). There is a cessation of literature that describes the impact of each SVI theme on CDI outcomes. The objective of this study was to utilize the four themes of the CDC SVI to determine the impact of SDoH on initial CDI severity and all-cause mortality outcomes.

**Methods:**

Adult patients admitted to Loma Linda University Medical Center (LLUMC) with an initial episode of CDI between January 2020 to June 2021 were included. Patient residential addresses were collected and input into the CDC/ATSDR SVI Interactive Map. Vulnerability groups were congregated into 2 main subgroups [patients with SVI scores of < 0.4999 are grouped as low vulnerability (LV) and patients with SVI scores of ≥ 0.5 are grouped as high vulnerability (HV)].

**Figure:**
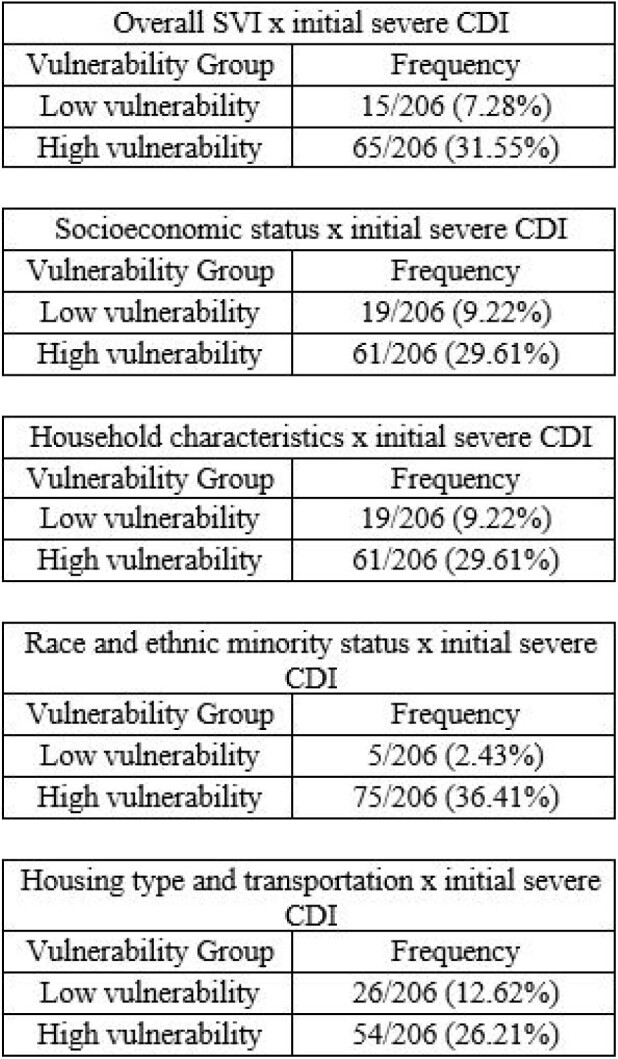

**Results:**

206 patients were included, and those with an overall HV SVI score were more likely to have a higher all-cause mortality rate and to present with severe or fulminant initial CDI compared to those with a LV score. The frequency of initial severe CDI was 3x greater for the SVI themes of SES and H&C [61/206 (29.61%) vs 19/206 (9.22%)] in patients with a HV score compared to those with LV scoring. Further, patients who presented with an HV score for the H&T theme were more likely to present with initial severe (26.21% vs 12.62%) or fulminant CDI (13.11% vs 5.34%) versus those with a LV score. When considering the REM status theme, the initial fulminant CDI diagnosis was about 37x greater among individuals with a HV score (17.96% vs 0.49%) and mortality 9x greater when compared to those with LV scoring (8.74% vs 0.97%).

**Conclusion:**

HV scores across SES, H&C, REM status, and H&T correlate with increased CDI severity and mortality rates. Further research and targeted interventions should be identified.

**Disclosures:**

**Anna Zhou, PharmD**, Melinta Therapeutics: Honoraria **Jacinda C. Abdul-Mutakabbir, PharmD, MPH**, CSL Sequiris: Advisor/Consultant|CSL Sequiris: Honoraria|GSK: Advisor/Consultant|GSK: Honoraria|Shionogi: Advisor/Consultant|Shionogi: Honoraria

